# CRISPR/Cas9-Based Genome Editing of Fall Armyworm (*Spodoptera frugiperda*): Progress and Prospects

**DOI:** 10.3390/biom14091074

**Published:** 2024-08-27

**Authors:** Yussuf Mohamed Salum, Anyuan Yin, Uroosa Zaheer, Yuanyuan Liu, Yi Guo, Weiyi He

**Affiliations:** 1 State Key Laboratory for Ecological Pest Control of Fujian and Taiwan Crops, Institute of Applied Ecology, International Joint Research Laboratory of Ecological Pest Control, Ministry of Education, Ministerial and Provincial Joint Innovation Centre for Safety Production of Cross-Strait Crops, Fujian Agriculture and Forestry University, Fuzhou 350002, China; yussufbedi.ym@gmail.com (Y.M.S.); 5220231036@fafu.edu.cn (A.Y.); uroosazaheer58@gmail.com (U.Z.); yuanyuanliu@fafu.edu.cn (Y.L.); 2 Plant Protection Research Institute, Guangdong Academy of Agricultural Sciences, Key Laboratory of Green Prevention and Control on Fruits and Vegetables in South China Ministry of Agriculture and Rural Affairs, Guangdong Provincial Key Laboratory of High Technology for Plant Protection, Guangzhou 510640, China

**Keywords:** fall armyworm, genome editing, gene function, pest control, sterile insect technique

## Abstract

The fall armyworm (*Spodoptera frugiperda*) poses a substantial threat to many important crops worldwide, emphasizing the need to develop and implement advanced technologies for effective pest control. CRISPR/Cas9, derived from the bacterial adaptive immune system, is a prominent tool used for genome editing in living organisms. Due to its high specificity and adaptability, the CRISPR/Cas9 system has been used in various functional gene studies through gene knockout and applied in research to engineer phenotypes that may cause economical losses. The practical application of CRISPR/Cas9 in diverse insect orders has also provided opportunities for developing strategies for genetic pest control, such as gene drive and the precision-guided sterile insect technique (pgSIT). In this review, a comprehensive overview of the recent progress in the application of the CRISPR/Cas9 system for functional gene studies in *S. frugiperda* is presented. We outline the fundamental principles of applying CRISPR/Cas9 in *S. frugiperda* through embryonic microinjection and highlight the application of CRISPR/Cas9 in the study of genes associated with diverse biological aspects, including body color, insecticide resistance, olfactory behavior, sex determination, development, and RNAi. The ability of CRISPR/Cas9 technology to induce sterility, disrupt developmental stages, and influence mating behaviors illustrates its comprehensive roles in pest management strategies. Furthermore, this review addresses the limitations of the CRISPR/Cas9 system in studying gene function in *S. frugiperda* and explores its future potential as a promising tool for controlling this insect pest.

## 1. Introduction

The genome editing technology of Clustered Regularly Interspaced Short Palindromic Repeats (CRISPR)/CRISPR-associated protein 9 (Cas9) originates from bacterial and archaeal immune systems, which play essential roles in defensive mechanisms against mobile genetic elements [1]. The CRISPR/Cas9 system has two main components: the Cas9 protein, which cleaves double-stranded DNA (dsDNA) to facilitate genome modification, and a chimeric 20-bp single-guide RNA (sgRNA), which guides Cas9 to identify and recognize the desired sequence [2,3]. The cleaved dsDNA can be repaired either by the mechanism of non-homologous end joining (NHEJ) or that of homology-directed repair (HDR) [4,5]. When the coding sequence (CDS) of the desired gene is targeted by CRISPR/Cas9 and repaired via NHEJ, which is an error-prone process, the insertions or deletions of random base pairs may result in the disruption of the CDS and, thus, nonfunctional protein or no protein at all. Meanwhile, HDR can generate mutations through integrating the exogenous genes of interest, which is called gene knock-in [6]. In the CRISPR/Cas9 system, sometimes, multiple sgRNAs are required to simultaneously target different loci for both the genes of interest and the marker genes that create visible phenotypes. This phenomenon, known as Co-CRISPR/Cas9, enhances the editing efficiency, since it allows for more accessible and faster identification of genomic modifications [7,8]. The CRISPR/Cas9 system is a versatile and powerful tool for genome editing that has been successfully applied across a wide range of organisms, including bacteria, plants, animals, and humans [9,10], involving various research fields, including agriculture, medicine, and environmental sciences [11,12]. 

Recently, CRISPR/Cas9 was used in the functional study of genes from different insects to evaluate the feasibility of its further application in developing an efficient tool for pest control [13]. For example, the *white* gene has been utilized as a phenotypic marker in a variety of insect orders. Mutations of this gene result in a distinctive and easily observable change into a white eye color, making it an effective and versatile marker for genome editing studies across diverse insect species [14,15]. Moreover, CRISPR/Cas9 has been used to study the function of the doublesex (*dsx*) gene through gene knockout, which is essential for reproduction and sexual dimorphism in many insects [16,17,18]. Therefore, the CRISPR/Cas9 system has potentially promising applications for studying gene functions, and could also be an essential tool for insect pest control through the introduction of genetic modifications that may affect adult fertility [6,19,20,21]. 

There are several versatile CRISPR/Cas9-based strategies, including gene drive, which promotes the super-Mendelian inheritance of the genes determining female fertility or viability; sex ratio distortion, which explicitly targets and disrupts the female sex chromosome; and the precision-guided sterile insect technique (pgSIT), which confers female-specific lethality and male-specific sterility by crossing different genetically engineered strains [6]. Due to its high specificity and adaptability, the CRISPR/Cas9 system has been used in the application of gene drive strategy for the control of malaria mosquito vectors, which are deadly to human life and costly for governments and out-of-pocket spending [22]. This could be achieved by driving maleness for the eradication of the targeted *Anopheles* species or the persistent inheritance of specific genes in the mosquitoes for obstructing the transmission of the Plasmodium parasite [23,24], which has wide implications for the control of agricultural insect pests that have caused significant economic losses [6].

The fall armyworm (FAW), *Spodoptera frugiperda* (JE Smith), is reported to originate from the American continent and has invaded more than 100 countries worldwide since 2016 due to its strong capacity for migration and reproduction [25]. It is a highly polyphagous pest that is capable of feeding on over 350 host plant species, including wheat, sorghum, maize, cowpea, rice, cotton, groundnut, soybean, and millet [26,27]. This species has been divided into two genetic strains: a rice strain that feeds on rice, sorghum, and soybean, and a corn strain that mainly feeds on maize [28]. In early 2016, *S. frugiperda* was reported in different African countries such as Nigeria, Togo, Ghana, Mozambique, Botswana, Zimbabwe, Kenya, Tanzania, Uganda, Ethiopia, and Benin [29,30,31]. During 2018 and 2019, the pest was discovered in many Asian countries, including China, Japan, India, South Korea, Yemen, Bangladesh, Thailand, Myanmar, Malaysia, Vietnam, and Singapore [29,32]. Various techniques have been used to control *S. frugiperda*, including the use of microorganisms and beneficial insects for biological control of the pest, the push–pull technique and inter-cropping systems for ecological control, and the use of synthetic insecticides for chemical control. Additionally, other techniques are recommended for the biosafety and effective suppression of the pest, including genetic control methods such as the sterilized insect technique (SIT), the development of transgenic plants, and genome-editing-based technology [27,33,34].

The advances in genome editing technology have led to an increased usage of CRISPR/Cas9 technology for the functional study of different genes in *S. frugiperda*, opening new avenues for exploring the genetic underpinnings of its biology. Furthermore, the progress made in CRISPR/Cas9 has significant prospects for developing innovative and sustainable pest control methods. By enabling precise genetic modifications, CRISPR/Cas9 holds the potential to create new strains with an enhanced susceptibility to biological control agents or reduced fitness in natural populations, thereby contributing to integrated pest management (IPM) strategies [10]. However, comprehensive reviews covering genome editing technologies using CRISPR/Cas9 in *S. frugiperda* are still limited. In this review, we provide an in-depth and comprehensive summary of the applications of CRISPR/Cas9 technology in the functional study of various biological aspects of *S. frugiperda*, such as those associated with body color, insecticide resistance, sex determination, development, olfactory behavior, and the RNAi machinery. CRISPR/Cas9-based technology introduces innovative strategies that could have significant implications for pest control and serve as effective tools for the sustainable management of this pest.

## 2. Procedure for Embryonic Microinjection

### 2.1. In Silico Design, In Vitro Synthesis, and Assessment of sgRNA

**Figure 1:**
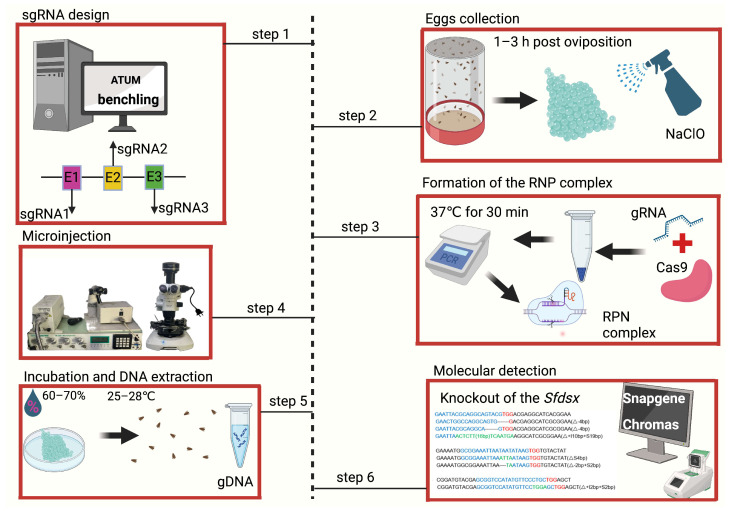
Overview of the processes for CRISP/Cas9-based genome editing in *S. frugiperda.* Step 1: sgRNA design. sgRNAs are designed using online tools, such as ATUM or benchling, targeting specific exons (E1, E2, and E3) of the gene of interest. Step 2: Formation of the RNP complex. The sgRNAs are mixed with the Cas9 protein, which is then incubated at 37 °C for 30 min, to form the RNP complex. Step 3: Egg collection. Eggs are collected from mated female adults within 1–3 h post oviposition and treated with NaClO for sterilization. Step 4: Microinjection. The prepared RNP complex is injected directly into the collected eggs using a specialized microinjector setup. Step 5: Incubation and DNA extraction. The injected eggs are incubated at 25–28 °C with a humidity of 60–70% for further development. The gDNA is extracted from the adults. Step 6: Molecular detection. The PCR product of the gDNA is analyzed through Sanger sequencing. By comparison with the wild-type sequence, any deletions or insertions in the individuals subject to microinjection are detected to confirm the success of the editing event [17].

The first step is to design and synthesize sgRNA, which focuses on the target site in the exon following the 5′-GG-(N)_18_-NGG-3′ criteria [17]. This sgRNA should typically be designed near the start of a gene, ideally within the first exon, with a GC content between 40 and 60%, avoiding regions with complex secondary structures that might hinder the binding of the sgRNA to target DNA [35]. This can increase the likelihood of disrupting gene function and enhance the stability and binding efficiency of the sgRNA, while avoiding the issues of the sgRNA binding to nontarget DNA (Figure 1, step 1).

It is important to design multiple sgRNAs targeting different regions of the gene to account for variability in cleavage efficiency. This strategy ensures that, if one sgRNA fails to induce efficient cleavage, others might still work effectively, saving time and resources. Tools like ATUM (https://www.atum.bio/eCommerce/cas9/input (accessed on 25 August 2024)) and benchling (https://www.benchling.com/crispr (accessed on 25 August 2024)) facilitate this design process by offering user-friendly interfaces for designing sgRNAs, providing detailed information on potential off-target sites and cleavage efficiency, and making comprehensive analyses and scores of potential sgRNA targets. sgRNA with a high score is better for minimizing the likelihood of editing a nontarget region [36].

Several commercial companies provide services for synthetic sgRNAs, including Synthego (www.synthego.com (accessed on 25 August 2024)), GENEWIZ (https://www.genewiz.com/ (accessed on 25 August 2024)), and Integrated DNA Technologies (https://sg.idtdna.com/pages (accessed on 25 August 2024)). GENEWIZ and Integrated DNA Technologies also provide commercial kits that are generally more stable, with consistent quality and a reduced risk of sgRNA degradation. However, they can be more expensive and require rigorous quality control and handling. After in silico design, the sgRNA is transcribed in vitro using a T7 promoter and stored at −80 °C until it is used for injection. The last and vital step is to validate and confirm the sgRNA activity in vivo [37].

### 2.2. Egg Collection and Preparation

Eggs are typically collected from substrates like leaves, paper towels, or container walls, where female adults may lay eggs. The choice of substrate can influence the egg-laying behavior, so it is recommended to mimic natural conditions as closely as possible. In rearing *S. frugiperda*, the male–female ratio plays a significant role in egg production. Males and females are placed together in a container to facilitate mating and subsequent egg laying. While a 1:1 ratio is typically used, slight variations can be tested to find the optimal ratio under different rearing conditions, providing control over the process for improving efficiency [38]. It is important to provide a suitable environment. Different containers, from small cages to large enclosures, can be used. However, regardless of size, the rearing setup must have adequate ventilation and space for movement to ensure the well-being of insects (Figure 1, step 2). A photoperiod of 16 h of light and 8 h of darkness is commonly used for rearing *S. frugiperda*. For the optimal egg-laying activity of the insects, humidity should be maintained at 60–70% and temperature should be kept at 25–28 °C [27]. Adults are fed with a sugar solution to ensure adequate nutrition. 

Extensive studies have revealed that, upon oviposition, the eggs should be collected within a narrow window of 1–3 h to maximize the chance of obtaining mutants [39,40]. The collected eggs are washed and treated with a 1% sodium hypochlorite (NaClO) solution to sterilize and remove unwanted debris [40,41]. Some studies suggest that distilled water is sufficient for cleaning the eggs before injection [10]. However, other experts believe the eggs should be injected immediately after collection without prior washing [42].

### 2.3. Microinjection

Microinjection, a pivotal step in CRISPR/Cas9 genome editing, demands the utmost precision and technical skill. It involves the direct delivery of the Cas9 and sgRNA mixture (ribonucleoprotein (RNP)) into the embryo for precise targeting of the desired gene. However, the high mortality rate caused by microinjection necessitates using a large number of eggs. Additionally, the injection process also requires specialized equipment and materials, making it resource-intensive.

In many CRISPR/Cas9 studies, RNP is commonly used for injection. However, a strategy of using plasmids that encode Cas9 and sgRNA or Cas9 mRNA along with the sgRNA can be an alternative, reducing the chances of off-target effects compared to using plasmids but requiring careful handling to avoid degradation [43]. In some studies, multiple sgRNAs targeting different sites are used for increasing the chance of editing events [35,36]. The concentration ratio of Cas9 to sgRNA typically used is 1:1 or slightly higher for Cas9. Various studies use different concentrations of the Cas9 protein and sgRNA, ranging from 100 to 500 ng/µL and 100 to 400 ng/µL, respectively, with approximately 1 nl injected for each egg [40,41,44,45]. These findings demonstrate that different mixture concentrations of Cas9 and sgRNA result in different egg-hatching rates in *S. frugiperda* (Table 1). Moreover, the hatching rate for eggs injected with RNP was significantly lower, ranging from 24% to 64%, compared to 80–90% of that observed in the uninjected control group. Therefore, sufficient eggs need to be injected in the RNP group to increase the number of surviving individuals [17,44].

Before injection, the Cas9 and sgRNA components are incubated at 37 °C for 30 min to form the RNP complex (Figure 1, step 3). The Cas9 proteins can be sourced from companies like New England Biolabs (https://www.neb.com/en (accessed on 25 August 2024)), Thermo Fisher Scientific (https://www.thermofisher.cn/cn/zh/home.html (accessed on 25 August 2024)), and Integrated DNA Technologies (https://sg.idtdna.com/pages (accessed on 25 August 2024)). They are stored at −20 °C or −80 °C. The different tools used in microinjection include a microscope, injection needles, and a microinjector (Figure 1, step 4). The needles should have a fine tip (approximately 0.5–1 µm) to penetrate the eggs without causing excessive damage. Also, the needles should be sharp and properly calibrated to ensure the precise delivery of the sgRNA–Cas9 mixture. Before starting the injection, the needles must be opened to allow the RNP to enter the embryo. Compressed air or nitrogen is needed to control the pressure applied during microinjection. The pressure used for injection is usually set between 20 and 60 pounds per square inch (psi), but it may require adjustment based on the egg types and needle specifications [49].

During the injection process, eggs are arranged in a way that provides easy access for the injection needle. Typically, the posterior end of the egg, where the pole cells begin to develop, is targeted for injection [50]. Therefore, orienting the eggs with this end facing up can facilitate the process. Eggs can be secured using double-sided tape or a specialized holding device to prevent movement during injection. According to the literature, the injection of 100–500 eggs is recommended to ensure a sufficient number of successfully edited embryos, considering the high mortality rates and variable success rates of individual injections. Editing efficiency can vary, but achieving at least 1–5% of injected eggs is an expected outcome [40,42]. It is crucial to maintain detailed records of the injection process, including the number of eggs injected and the volume of the sgRNA–Cas9 mixture used. These are invaluable for future reference and contribute significantly to advancing scientific knowledge in this field.

### 2.4. Incubation and Molecular Detection

The final step in embryonic microinjection is incubation, during which, the injected eggs need to be kept at specific temperatures and humidity levels, approximately 25–28 °C and 60–70%, respectively, for egg hatching and development [41,46]. Usually, it takes 2–5 d for eggs to hatch if the environmental conditions are suitable (Figure 1, step 5). 

After the insects have developed, genomic DNA is extracted from the edited individuals to assess the efficiency of the genome editing. A traditional method involves using phenol–chloroform extraction, which includes lysing cells, separating proteins and lipids from DNA, and purifying the DNA. Alternatively, commercial kits like the Qiagen DNeasy Blood & Tissue Kit (https://www.qiagen.com/ja-jp (accessed on 25 August 2024)) can be used, which are user-friendly and often provide high DNA yields and purity.

After DNA extraction, PCR is used to amplify the target region of the gene of interest. The PCR products are sequenced using the Sanger sequencing platform. The resulting chromatograms are processed and analyzed for insertions or deletions at the target site by comparison with the reference sequences using software like Chromas (https://technelysium.com.au/wp/ (accessed on 25 August 2024)), Geneious (https://www.geneious.com/ (accessed on 25 August 2024)), or SnapGene (https://www.snapgene.com/ (accessed on 25 August 2024)) (Figure 1, step 6).

Additionally, the T7 endonuclease I (T7E1) assay can be used to assess the efficiency of genome editing. The T7E1 cleaves mismatched DNA, indicating the presence of mutations. The cleavage products are analyzed by agarose gel electrophoresis, where the intensity of the cleaved bands compared to the untreated PCR product provides a quantitative measure of the editing efficiency.

## 3. Application of CRISPR/Cas9 Genome Editing in *S. frugiperda*

The microinjection of CRISPR/Cas9 components is the primary method for achieving precise genetic modifications in *S. frugiperda*. This precise genomic manipulation provides a powerful platform for investigating gene function and developing novel pest management strategies. The ability to efficiently edit the *S. frugiperda* genome has opened up new avenues for research in various biological areas, including body pigmentation, insecticide resistance, olfactory behavior, sex determination, and developments. Additionally, this technology enhances our understanding of the RNAi machinery and contributes to developing innovative solutions for the management of this insect pest.

### 3.1. Genome Editing Associated with Body Color

Genome editing techniques such as CRISPR/Cas9 have revolutionized the study of insect pigmentation by enabling targeted modifications in the key genes responsible for coloration. In particular, genes like kynurenine 3-monooxygenase (*kmo*), *ebony*, *scarlet*, and *yellow-y* play crucial roles in determining the body and eye color of many insects. Understanding the functions of these genes in *S. frugiperda* would shed light on the intricate mechanisms governing pigmentation across different developmental stages and species of lepidopteran insects.

One of the genes associated with color change in *S. frugiperda* is the kynurenine 3-monooxygenase (*kmo*) gene, which is responsible for eye and body pigmentation in many insects [51,52]. The *kmo* gene is most highly expressed at the adult stage of lepidopteran insects [52] and is essential in producing the xanthommatin pigment [51]. The knockout of *kmo* in *B. mori* results in white-colored eyes and eggs, different from the wild type with black eyes and blue eggs [52]. In *S. frugiperda*, the *kmo* gene is most highly expressed in the larval and adult stages, and the knockout of *kmo* in *S. frugiperda* resulted in a dark brown color of the larval body instead of the wild-type brown, with mutant adult moths showing mosaic eyes. Additionally, mutant eggs appeared emerald compared to those of the wild-type brown [53].

The *ebony* and *scarlet* genes play significant roles in controlling the eye and body coloration in *S. frugiperda*, with *ebony* specifically regulating the production of the black melanin pigment [54]. The *ebony* gene encodes a synthetase that produces N-β-alanyl-dopamine (NBAD) from dopamine, suppressing melanin during cuticle development. A deficiency in the *ebony* gene can dramatically alter pigmentation levels, leading to a deep black coloration [54,55,56]. The knockout of the *ebony* gene in *S. litura* resulted in deep coloration in mutant pupae compared to wild-type pupae [55]. In *P. xylostella*, the knockout of the ebony gene led to dark coloration in larvae, pupae, and adults compared to wild-type individuals. Additionally, in the larval stage, the heads and legs of mutants were black instead of the wild-type brown [57]. In *S. frugiperda*, the knockout of the *ebony* gene caused distinct body color changes, with the pupae and adults displaying a deep black color instead of the wild-type light brown [58].

The *scarlet* gene regulates eye pigmentation in insects, and its absence leads to altered eye coloration [59]. This gene encodes a protein facilitating the transportation of pigments into eye granules [59]. *Scarlet* gene expression reaches its peak during the pupal and adult stages in many lepidopteran insects [60]. Knockout effects of the *scarlet* gene have been observed in other lepidopteran insects, such as *B. mori* and *Helicoverpa armigera* mutants exhibiting white eyes instead of the wild-type black and striking yellow compound eyes instead of the wild-type green, respectively [59,61]. In *S. frugiperda*, the knockout of the *scarlet gene* results in bright yellow eyes instead of the wild-type dark brown [58]. 

The *yellow-y* gene is crucial for insect body coloration, generating black melanin in lepidopterans like *P. xylostella*, *B. mori*, *S. litura*, and *Agrotis ipsilon* [62,63,64,65]. This gene is vital for melanin production, and its deficiency reduces black melanin, shifting the body color from black to yellow [54]. Some lepidopteran insects express *yellow-y* highly in their larval and adult stages [63,66]. The knockout of the *yellow-y* genes in *P. xylostella* and *A. ipsilon* resulted in yellow bodies at the larval stage compared to the wild types with light-black- and black-colored bodies, respectively [63,65]. In *S. frugiperda*, *yellow-y* is predominantly expressed in the larval and pupal stages [37]. The knockout of the *yellow-y* gene in *S. frugiperda* resulted in mutant larvae displaying yellow-brown heads and bodies, differing from the wild-type brown. At the same time, mutant adults exhibited yellowish wings, legs, and bodies [37].

By studying these pigmentation genes, researchers can understand the mechanisms underlying pigments’ production across insects’ development stages. These genes are highly effective markers in knock-in experiments due to their easily observable and non-invasive phenotypes. This knowledge holds promise for applications in pest management based on genome editing.

### 3.2. Genome Editing Associated with Insecticide Resistance 

The use of chemical insecticides for pest control has increased, although their target spectrum is general rather than specific, causing potential threats to biosafety. However, this practice contributes to the developing resistance among pests, posing a significant risk to agricultural sustainability. ATP-binding cassette (*ABC*) transporters play crucial roles in resistance formation in many insects [67,68,69]. *ABC* transporters facilitate the translocation of molecules across membranes via ATP binding [70]. 

In many lepidopteran insects, *ABCB1* is expressed mainly in the larval and adult stages and is associated with pesticide resistance, with a higher expression occurring in resistant strains [71]. In *S. exigua*, *ABCB1* knockout increased their sensitivity to abamectin and emamectin benzoate [72]. In *S. frugiperda*, the expression pattern of the *ABCB1* gene is still unknown. However, the CRISPR/Cas9-based knockout of *ABCB1* in *S. frugiperda* increased its susceptibility to emamectin benzoate, beta-cypermethrin, and chlorantraniliprole [47].

ABCC2 and ABCC3 are other members of the ABC transporter family and are associated with chemical insecticide resistance, primarily expressed in the larval stage in many lepidopteran insects [73]. The knockout of *ABCC2* and *ABCC3* in another lepidopterans like *P. xylostella* causes increased resistance to Cry1Ac protoxin for both knockout strains, while in *Ostrinia furnacalis*, *ABCC2* knockout results in increased susceptibility to abamectin and chlorantraniliprole [74,75]. The knockout of each of these two genes in *S. frugiperda* via CRISPR/Cas9 increased its susceptibility to abamectin and spinosad. The *ABCC2* knockout strains exhibited susceptibilities against abamectin and spinosad that were 7.8-fold and 3.1-fold higher, respectively, while *ABCC3* strains showed susceptibilities that were 4.5-fold and 2-fold higher, respectively [76].

Nicotinic acetylcholine receptors (*nAChR*s) function as ligand-gated ion channels targeted by insecticides like spinosad, dinotefuran, and spinetoram [77,78]. Mutation of the gene coding for the *nAChR α6 subunit* (*nAChRα6*) contributes to insecticide resistance in various lepidopteran pests, including *Drosophila melanogaster*, *P. xylostella*, *Tuta absoluta*, *H. armigera*, and *S. exigua* [79,80,81,82]. In *P. xylostella*, the knockout of the *nAChRα6* gene increased its resistances by 229- and 1462-fold against spinosad and spinetoram, respectively, while in *H. armigera*, the knockout strains showed increased resistances by 373- and 850-fold for spinosad and spinetoram, respectively [80,81]. In *S. frugiperda*, the knockout of *nAChRα6* conferred significantly higher resistance to spinosad and spinetoram by 307- and 517-fold, respectively [46]. 

In a recent study, CRISPR/Cas9 was used to verify the resistance in *S. frugiperda* to the insecticidal protein Vip3Aa from *Bacillus thuringiensis* (Bt), which was caused by the retrotransposon-mediated alternative splicing of a midgut-specific chitin synthase (*CHS2*) gene [83].

Studying the genes associated with insecticide resistance in insect pests provides a practical solution to combat the growing resistance challenge in many agricultural pest species. Understanding and targeting these specific genes can help to disrupt the genetic pathways that confer resistance. Implementing strategies based on genome editing can lead to more sustainable pest control measures by reducing the usage of chemicals.

### 3.3. Genome Editing Associated with Olfactory Behavior 

Many insects rely on olfaction for vital activities like feeding, mating, defense, and reproduction [84]; this could be an ideal target for developing management strategies, such as trapping male pests using sex pheromones [85]. The *Orco* gene is pivotal in insect olfaction, governing behavior like mating, egg laying, food recognition, and host plant localization [86]. Orco is part of the insect odorant receptor complex, which fulfils several functions, including transporting the receptor complex to the cell membrane after synthesis and maintaining the structural integrity of the receptor in the membrane, as well as transporting cations of Na^+^, K^+^, and Ca^2+^ [87]. The *Orco* gene is predominantly expressed in larvae and adults across many insect species [88]. The knockout of *Orco* in *B. mori* causes male homozygous mutants to display no response to sex pheromones [89], while male *Orco* mutants in *M. sexta* and *H. armigera* could not recognize females, attenuating their reproductive ability [90,91]. In *S. frugiperda*, the *Orco* gene is highly expressed in the adult antennae of both sexes compared to the other organs [42]. The knockout of the *Orco* gene in *S. frugiperda* drastically reduced the male adult response to sex pheromones and affected mating behavior [42]. Further, the mutation of *Orco* also affected the feeding behavior of *S. frugiperda*, with mutant larvae taking significantly longer to detect food than wild-type larvae [42]. Similarly, in *B. mori* and *H. armigera*, *Orco* mutant larvae showed reduced chemotaxis activity towards *cis*-jasmone and green peppers, respectively [91,92].

Another gene associated with olfactory behavior in *S. frugiperda* is the odorant-binding protein 27 (*OBP27*). Odorant-binding proteins (OBPs) are small and water-soluble proteins that facilitate the transport of odorant molecules to their relevant receptors across the sensillum lymph to the dendrite membrane [93,94]. The function of this gene varies among insect species. For example, in *S. frugiperda*, *OBP27* is vital for reproduction, host adaptation, and development, while in *Athetis dissimilis*, OBPs are crucial for reproduction [39,95]. The *OBP27* genes of lepidopterans, such as *S. litura*, *A. dissmilis*, and *H. armigera*, are expressed mainly in the antennae [95,96,97]. In *S. frugiperda*, *OBP27* expression is prominent in the fat body and male adult reproductive organs [39]. The knockout of *OBP27* in *S. frugiperda* led to longer larval development, reduced larval weight, and altered mating behavior, with mutant pairs exhibiting lower success rates and longer mating durations than the wild type [39].

A recent study on the olfactory receptors in *S. frugiperda* focused on ionotropic receptors (IRs), which detect acids, amines, and aldehydes [98] and contribute significantly to insect food and taste assessment [99]. Most IRs are expressed on the antennae part of lepidopteran insects for the function of acid detection [100,101,102]. The knockout of the *IR8a* gene in *Agrotis segetum*, *M. sexta*, and *H. armigera* exhibited repellent behavior in the mutants to octanoic acid and aldehyde, confirming its involvement in the response to acidic stimuli [101]. In *S. frugiperda*, the gene *IR75q.2* is expressed mainly on the antennae part of the adult, and the knockout of *IR75q.2* reduced adult responses to acids and aldehydes. Additionally, oviposition decreased by 50% in mutants compared to the wild type [103].

When infested by insect herbivores, plants can emit a compound of herbivore-induced plant volatiles (HIPVs), which regulate plant defenses and even herbivore immunity to parasitoids [104]. The glucose oxidase (GOX) of caterpillar has also been well-known to modulate plant defenses [105]. A GOX homolog (*SfruGOX*) that is highly expressed in the larval salivary gland was identified in *S. frugiperda*. A knockout experiment on *SfruGOX* using CRISPR/Cas9 revealed its roles in suppressing at least three maize (*Zea mays*) green leaf volatiles while eliciting terpenoid production [106], implying a close association of feeding behavior with the HIPVs mediated by the insect olfactory system. 

Moreover, CRISPR/Cas9 techniques were employed to investigate the role of the ∆11-desaturase 1 gene (*SfruDES1*) in *S. frugiperda*. This gene is associated with the biosynthesis of female sex pheromones in different lepidopterans, which is crucial for sexual communication and reproductive behavior based on the olfactory system [107,108,109]. In lepidopterans, the gene is expressed mainly in female adults, especially in the sex pheromone glands [108,110]. In *S. frugiperda*, this gene has a high expression in the female sex pheromone gland. The knockout of *SfruDES1* led to decreased mating behavior and attractiveness of mutant females to males, and a decrease in the number of eggs produced by mutant females compared with the wild type [109]. Meanwhile, the CRISPR/Cas9-based mutation of another essential gene, *SfruDES9*, in the pheromone biosynthesis of *S. frugiperda* can also cause mating disruption in female adults [111]. Further, when the upstream regulator, the pheromone biosynthesis-activating neuropeptide (*PBAN*) gene, was knocked out using CRISPR/Cas9, the mutant female adults became less attractive than the wild type, and showed no fecundity [112].

Understanding the olfactory behavior of *S. frugiperda* provides valuable insights into how this pest detects and responds to environmental cues. This knowledge can help in the design of effective attractants or repellents and enhance pest monitoring and control programs. By disrupting olfactory pathways, based on genome editing strategies, related to crucial behaviors such as mating, foraging, and oviposition, we can effectively reduce pest populations and minimize crop damage, improving agricultural productivity and the ecological balance.

### 3.4. Genome Editing Associated with Sex Determination

Sexual determination is a crucial biological process determining the development of sexual characteristics in insects, directly impacting their reproductive capabilities. Insect sex determination involves a cascade of genetic and molecular mechanisms, which can vary significantly across different species [6]. Understanding these mechanisms has opened up possibilities for genetic pest control, which aims to manage pest populations by interfering with their reproductive processes. 

One of the critical genes involved in this process is the sex-lethal (*sxl*) gene. This gene is essential in sex determination and reproduction across various insect species [113]. In Lepidoptera, the *sxl* gene plays a significant role, particularly in producing and migrating pyrene and eupyrene spermatozoa in the female reproductive tract [114]. The *sxl* gene encodes a protein responsible for male sperm production and assisting egg migration in females [115]. It is primarily expressed in the sex organs of both male and female insects [48,116]. The knockout of *sxl* in *B. mori* led to complete sperm motility loss and reduced male reproduction rates, while in *S. litura*, the mutation of *sxl* resulted in reduced fecundity and egg-hatching rates [113,116]. In *S. frugiperda*, the *sxl* gene is also expressed mainly in the sex organs, and the knockout of the *sxl* gene resulted in sterility, with a zero hatching rate observed when mutant males or females mated with wild-type insects of the opposite sex [48]. 

The other gene for *S. frugiperda* sex determination is the *dsx* gene. In most insect species, male and female differentiation is primarily driven by the *dsx* gene, the pre-mRNA of which undergoes sexually dimorphic alternative splicing to yield female- and male-specific isoforms (*dsxF* and *dsxM*) [113]. The *dsx* gene is expressed during the early embryonic stages and persists until maturity to regulate the development of sexually dimorphic traits such as body size, abdominal genitalia, and sex-specific physiology [117,118]. The disruption of the *dsx* gene leads to improper sexual dimorphism in mutants, as observed in various lepidopteran insects such as *P. xylostella* and *O. furnacalis*, which show decreased fertility and fecundity [16,119]; this can serve as a tool for pest control [120]. In a knockout study of the *dsx* gene in the male-specific region (exon 5) in *S. frugiperda*, the mutant male contributed to a 91% reduction in the fecundity of a mated wild-type female, and the corresponding hatching rate of eggs was almost zero. In comparison, the mutant female exhibited an 80% reduction in fecundity, and the eggs were unable to hatch normally when knocked out in the female-specific region (exon 4). In addition, it was found that the mutation of the *dsx* gene affected the gene expression of olfactory receptor 1 (OR1), pheromone-binding protein 1 (PBP1), and pheromone-binding protein 2 (PBP2), which are associated with mating and oviposition, resulting in sterility for both male and female mutants. Observation showed that the three transcripts all decreased in mutants of the male adults, while they increased in mutants of the female adults compared to the wild type [17].

A detailed understanding of the genetic underpinnings of insect reproduction provides crucial insights into the mechanisms that govern their fertility and reproductive behavior. This knowledge is essential for applying pgSIT strategies to control pest populations by releasing genetically sterilized individuals into the field. By leveraging this genetic information, pgSIT strategies can offer more effective and long-term control of pest populations without relying on chemical insecticides. 

### 3.5. Genome Editing Associated with Development

Understanding the roles of essential developmental genes in insects is crucial for developing targeted pest control strategies. Insect development is regulated by genes influencing various physiological processes. These genes not only determine the physical characteristics and development of insects, but also impact their reproductive capabilities and survival [121]. This section will explore the functions and impacts of five critical genes in the development of *S. frugiperda*: the homeotic gene abdominal-A (*abd-A*), the hormone receptor 3 (*HR3*) gene, the prophenoloxidase (*PPO*) gene, the serine protease snake-like 1 (*SPSL1*) gene, and the testis-specific serine/threonine kinase 2 (*tssk2*) gene.

The homeotic gene abdominal-A (*abd-A*) plays a vital role in insect development, including abdominal segment determination, body axis differentiation, heart cell specification, nervous system and fat body development, gonadal genesis, midgut formation, and muscle patterning [122,123]. In many insect species, it is predominantly expressed in the egg, pupal, and adult stages [124]. The knockout of the *abd-A* gene in lepidopterans such as *P. xylostella* led to abdominal fusion and abnormalities, potentially causing sterility due to genitalia organ abnormalities [125,126]. Similarly, the knockout of this gene in *S. frugiperda* had severe consequences, resulting in fewer hatched eggs with discolorations and missing developmental organs. The mutant larvae exhibited abnormal features like a fused abdomen, often failing to reach the pupal stage, while adults failed to produce eggs [44]. 

Hormone receptor 3 (*HR3*) is a critical regulatory gene in the ecdysis process and a crucial factor in *S. frugiperda* development [127,128]. The *HR3* gene is expressed mainly in the larval stage for many insect species [129,130], predominantly in the prothoracic gland (PG), and is closely linked with molting and development [131]. In *S. frugiperda*, the *HR3* gene is mainly expressed during the embryonic and pupal stages, and its expression is predominant in the pupal PG [127]. The knockout of *HR3* in *S. frugiperda* using CRISPR/Cas9 technology produced undeveloped larvae that remained trapped within the eggshell. This observation serves as convincing evidence for the significant role played by the *HR3* gene in embryonic development [127]. 

Another gene involved in the developmental process of *S. frugiperda* is the prophenoloxidase (*PPO*) gene, which is crucial for various aspects such as immunity, detoxification, cuticle melanization, and molting [132]. During immune responses, *PPO*s catalyze the conversion of phenolic compounds of tyrosine into quinones, leading to pathogen elimination and melanin production, causing hemolymph darkening and cuticle blackening [133]. In many lepidopteran insects, the *PPO* genes are expressed mainly in the larval stage [134]. In *S. frugiperda*, two *PPO* genes were identified, *PPO1* and *PPO2* [135]. They are expressed in all developmental stages of *S. frugiperda*, with the highest expression occurring in sixth instar larvae [136]. The knockout of *PPO1* or *PPO2* resulted in smaller mutant pupae than the wild type, with *PPO1* mutants exhibiting high mortality rates and decreased adult survival rates [136].

The serine protease snake-like 1 (*SPSL1*) gene was observed to predominantly exist in the male reproductive tracts of *S. frugiperda*. The knockout of this gene based on CRISPR/Cas did not affect male spermatogenesis or sperm migration, but disrupted spermatophore formation and sperm activation after mating with female adults [137]. Furthermore, a testis-specific serine/threonine kinase 2 (*tssk2*) gene involved in spermatogenesis has been validated as the target gene for producing male sterility [138].

The exploration of key developmental genes in *S. frugiperda* reveals their crucial roles in various physiological processes, from embryonic development to adult reproduction and immunity. Disrupting these genes can lead to severe developmental abnormalities, reduced fecundity, and high mortality rates. These findings highlight their potential as targets for genetic pest control. By manipulating these genes based on genome editing strategies, researchers can develop an innovative method to control this pest effectively, promoting sustainable agricultural practices. 

### 3.6. Genome Editing Associated with RNAi Machinery 

RNA interference (RNAi) is a powerful genetic tool used to study gene function and develop pest control strategies. RNAi works by silencing specific genes through the introduction of double-stranded RNA (dsRNA) [139], which triggers the degradation of the target mRNA or prevents protein translation [140]. This mechanism is part of insects’ natural defense system against viruses and is crucial for regulating gene expression [141]. Two key components of the RNAi pathway in *S. frugiperda* are Dicer-2 and dsRNase. Understanding the roles and manipulation of these components can enhance the efficiency of RNAi-based strategies and improve pest management practices.

*Dicer-2* is essential in the insect defensive mechanism against the antiviral response that initiates the small interfering RNA (siRNA), which is complementary to viral RNA; it recognizes and cleaves double-stranded RNA (dsRNA), thus suppressing virus replication in insects [142]. The absence of this protein results in increased virus replication and insect infection [143]. In many lepidopteran insects, such as *B. mori*, *D. melanogaster*, and *H. armigera*, observation shows that the disruption of the *dicer-2* gene increased susceptibility to different viral infections [143,144,145,146]. In *S. frugiperda*, the knockout of the *dicer-2* gene by CRISPR/Cas9 showed an increase in the infection and replication of the virus by 1.5-fold compared with the parental *Sf9* cell line after five days of treatment with autographa californica multiple nucleopolyhedroviruses (AcMNPV) [147]. 

CRISPR/Cas9 has also been used to deactivate the dsRNase in *S. frugiperda*, which degrades dsRNA in insect midguts [148]. dsRNAase is an extracellular nuclease that harbors a conserved DNA/RNA non-specific nuclease domain [149,150]. It is prominently expressed in the intestinal tract, hemolymph, and head of the larval stage for different lepidopteran insects [151,152,153]. In *S. frugiperda*, it is prominently expressed in the insects’ digestive systems, and the knockout the *dsRNAase* gene enhances RNAi efficiency. This was proven through mutant larvae feeding on an artificial diet containing dsRNA of the *IAP* (inhibitor of apoptosis) gene, where the 37% mortality rate observed was higher than that for wild-type larvae [148].

By disrupting critical RNA degradation pathways, the efficacy of RNAi was significantly boosted, leading to higher mortality rates and reduced fitness among the targeted pest populations. Further research in this area using genome-editing-based strategies holds promise for refining and optimizing RNAi applications in pest management.

## 4. Limitation and Future Applications of CRISPR/Cas9-Based Strategies for the Control of *S. frugiperda*

**Figure 2:**
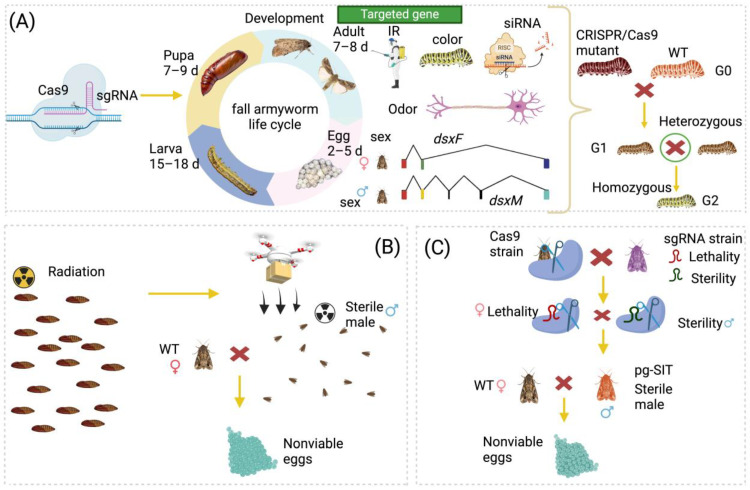
CRISPR/Cas9-based genome editing and future application in the control of *S. frugiperda*. (**A**) A pictorial presentation of the application of CRISPR/Cas9 in the knockout of genes associated with color, insecticide resistance, olfactory behavior, sex determination, development, and RNAi machinery. The heterozygous mutant created by CRISPR/Cas9 is crossed with the wild type for screening a homozygous knockout strain. *dsxF*, female splicing form of the doublesex gene; *dsxM*, male splicing form of the doublesex gene. (**B**) For the classical SIT used for the control of *S. frugiperda*, male pupae are irradiated, and sterile male adults are released into the field. These sterile males are mated with wild-type females, resulting in production of nonviable eggs. (**C**) For the potential application of pgSIT strategy, the *S. frugiperda* strain that expresses a functional Cas9 protein is crossed with another *S. frugiperda* strain that expresses two types of sgRNAs, which has a lethal effect on females and induces sterility in males. When the resulting pgSIT sterile males are mated with wild-type females, theoretically, nonviable eggs are produced. Together, these approaches have only been employed successfully in the laboratory and have shown a potential effectiveness in controlling *S. frugiperda*. Ongoing studies focus on large-scale field trials to validate the laboratory findings and represent innovative strategies for managing pest populations with the help of CRISPR/Cas9 technology.

CRISPR/Cas9 technology has been widely used in studying the genes associated with different biological functions in *S. frugiperda*, such as growth and development, sex determination, and reproduction, which will be potential targets for controlling the *S. frugiperda* population using molecular approaches (Figure 2A and Table 2). While Cas9 is the most widely used Cas enzyme for genome editing, recent advances have identified several other Cas enzymes, including Cas12a, Cas12b, Cas13, and Cas7-11 [154], which offer distinct advantages. These Cas enzymes have different PAM sites compared to Cas9. For instance, Cas12a and Cas12b recognize T-rich PAM sites (TTTVs), which would increase target site flexibility and genome editing effectiveness [155]. Cas13 targets RNA rather than DNA and does not require a PAM site [156]. Depending on the species, these alternative Cas enzymes generally produce fewer off-target effects and exhibit less cytotoxicity than Cas9. Additionally, the smaller size of these alternative Cas enzymes facilitates more efficient and effective delivery systems [154]. Altogether, these enzymes expand the diversity of the CRISPR/Cas genome editing tools available.

However, the application of CRISPR/Cas9 in *S. frugiperda* is hindered by several key challenges that researchers must address to improve its efficacy. The effective expression of Cas9 and sgRNAs necessitates strong, tissue-specific promoters. Several highly active promoters, such as *hr5/SfPub-P2009* and *hr5ie1* from *S. frugiperda*, have been identified and characterized in vivo [41,157]. Moreover, enhancers are required further to enhance the effective expression of Cas9 and sgRNAs. Identifying safe harbor sites in the genome for gene knock-in without disrupting essential genes is not just crucial, but a reassuring aspect of the process for *S. frugiperda*. The *rosy* locus in *Drosophila melanogaster* is a successful example of a safe harbor site for integration [158]. A significant consideration in CRISPR/Cas9 application is the temporary expression of Cas9 to minimize potential toxicity. Continuous Cas9 expression can be detrimental to the organism. To mitigate this, inducible promoters can be employed to control Cas9 expression at specific stages, organs, or ages [157]. Additionally, utilizing Cas enzymes with reduced off-target activities can help to minimize toxicity. For the successful population control of *S. frugiperda* using CRISPR/Cas9, it is essential and urgent to ensure that genome-edited individuals can successfully compete to mate in the wild. This requires evaluating the fitness and reproductive behavior of genetically edited strains under both laboratory and semi-field conditions. This ability is crucial for the long-term success of CRISPR/Cas9-based pest control strategies.

A common challenge for the application of CRISPR/Cas9 includes off-target effects, where the Cas9 enzyme inadvertently cuts DNA at unintended locations, leading to unpredictable mutations [159]. To mitigate this, researchers have developed improved Cas9 enzyme variants with a higher specificity, such as SpCas9-HF1 and xCas9, and employed computational tools to predict potential off-target sites. Another critical limitation lies in the efficient and effective delivery of CRISPR/Cas9. Microinjection, the primary delivery method, has several limitations: it requires specialized skills and is labor-intensive and unsuitable for large-scale genetic screening [160]. To address this, a significant portion of ongoing studies are dedicated to developing advanced in vitro delivery systems like viral vectors, lipid nanoparticles, and physical methods that could be used for the editing of Sf9 cell lines [147,161,162]. Meanwhile, some other in vivo delivery methods like direct parental CRISPR (DIPA-CRISPR) can also be explored [163]. Furthermore, the unpredictable nature of the DNA repair mechanism of HDR following Cas9-induced double-strand breaks poses a challenge. This mechanism can introduce unintended insertions or deletions, compromising the precision of the desired editing [164]. Additionally, designing sgRNAs that are both efficient and specific remains a hurdle. Bioinformatics tools and empirical testing have been employed to address this, but optimization continues to be a focal point [159]. While CRISPR/Cas9 offers promising prospects for pest control, overcoming challenges related to off-target effects, delivery efficiency, DNA repair mechanisms, and sgRNA design is imperative for its successful and widespread application. To this end, Co-CRISPR and multiple sgRNA/Cas9 methods have been used to simultaneously investigate the function of the marker genes associated with eye or body color and the target genes involved in the development of insecticide resistance in *S. frugiperda*, which have shown advantages in gene function studies [45,53].

The SIT is a well-established and environmentally friendly strategy crucial in pest management. This technique involves the mass rearing of target insect species, which are then sterilized using radiation methods such as X-rays, γ-rays, or electron beams [165]. This radiation induces chromosomal breakage, effectively sterilizing the insects. These SIT-modified insects could be used for release into the field, where they compete to mate with wild female adults, reducing the overall population over successive generations. The SIT has been successfully applied to control various lepidopteran pests, including *Lobesia botrana* and *Cydia pomonella* [166,167]. However, in *S. frugiperda*, a recent study revealed that 250 Gy radiation significantly reduced ovipositional and mating behavior, while complete sterility for both sexes was achieved at 400 Gy [33]. The potential negative impacts of radiation on insect fitness may limit the effectiveness of the SIT. 

CRISPR/Cas9 technology, a promising tool, can also be applied in developing genetic tools for pest control [6,10]. However, its application to *S. frugiperda* is largely limited [41]. Considering the relatively low efficiency of the gene drive system applied in lepidopteran insect pests based on the commonly used regulatory elements [6,168,169], we propose to use the pgSIT system as an alternative to the conventional SIT approach (Figure 2B). 

The pgSIT scheme is an effective scheme that relies on the establishment of an activator strain that can express a functional Cas9 protein and an effector strain that produces two types of sgRNAs for producing both female-lethal and male-sterile traits [6,120]. The progeny from the crossing between the two strains may only contain sterile males, which could be used in pest control, just as the sterile males from the conventional SIT program (Figure 2C). In *S. frugiperda*, the genes *SPSL1* and *tssk2* are candidates associated with the fertility of male adults [137,138]. Meanwhile, we also proposed the *dsx* and *sxl* genes to be potential target genes for the pgSIT (Figure 2B), as they can cause both lethality in females and sterility in males under the modulation of the CRISPR/Cas9 system [17,48]. 

As the field of insect molecular biology progresses, CRISPR/Cas9 technology has the potential to transform pest management by offering a safe and highly efficient solution. The use of CRISPR/Cas9 technology extends beyond traditional methods, enabling researchers to explore various pest management strategies at the genetic level and offering a more refined and sustainable approach for future applications in the control of *S. frugiperda*. This may also facilitate further development of the IPM by incorporating principles of synthetic biology in diverse agricultural settings.

## Figures and Tables

**Table 1:** Components and procedures of microinjection for CRISPR/Cas9 genome editing studies in *S. frugiperda*.

sgRNA and Cas9 Concentration (ng/µL)	Egg Collection Duration	Egg Preparation	Incubation Condition (Temperature and Humidity)	Hatching Percentage	Reference
100 and 300	1 h	1% sodium hypochlorite	27 ± 1 °C and 60%	62%	[45]
250 and 200	2 h	1% sodium hypochlorite	26 ± 1 °C and 65%	5.3%	[46]
250 and 500	3 h	1% sodium hypochlorite	26 ± 1 °C and 60%	67.2%	[44]
150 and 300	3 h	none	26 ± 1 °C and 70 ± 5%	14.9%	[42]
400 and 500	2 h	1% sodium hypochlorite	27 ± 1 °C and 65 ± 5%	32%	[39]
150 and 100	2 h	distilled water	25 °C and 65%	18.5%	[47]
400 and 500	1 h	distilled water	25 ± 2 °C, 70–80%	23.3%	[48]
300 and 300	1 h	none	25 °C	36.3%	[17]

**Table 2:** A list of functional genes of *S. frugiperda* that have been characterized with CRISPR/Cas9.

Gene of Interest	Gene Function	KO Phenotype	References
*Kynurenine-3-monooxygenase* *(kmo)*	Eye and body pigment production	Changes in body, eye, and egg colors	[53]
*ebony*	Melanin formation	Deep coloration in pupal and adult stages	[58]
*scarlet*	Transport pigment for eye color	Changes in color during pupal and adult stages	[58]
*Yellow-y*	Production of black melanin and black color	Yellow body color	[37]
*ATP-binding cassette B1/C2/C3 (ABCB1/ABCC2/ABCC3)*	Pesticides resistance	Increased susceptibility to emamectin benzoate, chlorantraniliprole, spinosad, and abamectin	[47,76]
*Nicotinic acetylcholine receptors* *(nAChR)α6*	Pesticides resistance	Increased resistance against spinosad and spinetoram	[46]
Chitin synthase (*CHS2*)	Resistance to insecticidal protein Vip3Aa	Increased resistance to Vip3Aa	[83]
*Odorant receptor co-receptor (Orco)*	Response to stimulus	Failures in adult responses to sex pheromones, predators, and food	[42]
*Odorant binding protein-27 (OBP27)*	Chemo-sensory recognition of stimulus	Prolonged larval duration, weight loss, and reduced mating behavior	[39]
*Ionotropic receptors* *(IR75q.2)*	Response to aldehyde and acid	Reduced responses to acids and aldehydes; also, oviposition behavior decreased	[103]
*Glucose oxidase (GOX)*	Suppression of plant defense against herbivore	Decreased green leaf volatile emission and increased terpene emission from the maize	[106]
*Doublesex (Dsx)*	Sexual dimorphism and determination	Infertility and decreased fecundity	[17]
*Delta11-desaturase1 (DES1)*	Biosynthesis of female sex pheromones	Decreased mating behavior and number of eggs produced by mutants	[109]
*Acyl-Co-A delat-9 desaturase (DES9)*	Biosynthesis of sex pheromone	Decreased mating behavior	[111]
*Pheromone biosynthesis activating neuropeptide (PBAN)*	Biosynthesis of sex pheromone	Decreased mating behavior and zero fecundity	[112]
*Sex-lethal (Sxl)*	Sex determination and reproduction	Reduced reproductive ability and induced sterility	[48]
*Prophenoloxidases (PPOs)*	Development and defense	Reduced pupal size and increased mortality	[136]
*Abdominal-A (Abd-A)*	Growth and development	Reduced hatching rate, failure in transition from larva to pupa, and sterility	[44]
*Testis specific serine/threonine kinase 2 (tssk2)*	Spermatogenesis	Male sterility	[138]
*Serine protease snake-like 1 (SPSL1)*	Regulation of spermatophore formation and sperm activation in female	Reduced fecundity rate	[137]
*Hormone receptor 3 (HR3)*	Growth and development	Molting failure in larvae and increased mortality	[127]
*Dicer-2* *(sf9 cell line)*	Defensive	Increased virus replication and infection	[147]
*Double-stranded RNAase (dsRNAase)*	Degrades dsRNA	Increases RNAi efficiency	[148]
